# Immuntherapien von Allergien: Aktueller Stand

**DOI:** 10.1007/s00103-020-03224-6

**Published:** 2020-11-02

**Authors:** Vera Mahler, Jörg Kleine-Tebbe, Stefan Vieths

**Affiliations:** 1 Paul-Ehrlich-Institut, Paul-Ehrlich-Str. 51–59, 63225 Langen, Deutschland; 2 Allergie- und Asthma-Zentrum Westend, Berlin, Deutschland

**Keywords:** AIT, Zulassungsverfahren, Therapieallergene-Verordnung, Wirkstoff, Individualrezeptur, AIT, Marketing authorization procedure, Therapy allergen ordinance, Active substance, Named patient product

## Abstract

Die Allergenimmuntherapie (AIT) wirkt als einzige kausale, krankheitsmodifizierende Therapieform neben einer Symptomlinderung der Progression allergischer Erkrankungen entgegen. Dieser Beitrag liefert einen Überblick zu aktuellen immunologischen, regulatorischen und praxisbezogenen Aspekten der AIT. Die neueste Fachliteratur wurde einbezogen. Zudem werden konzeptionelle regulatorische Entwicklungen aus der Abteilung Allergologie der zuständigen Bundesoberbehörde Paul-Ehrlich-Institut dargestellt.

Die derzeit in Deutschland zugelassenen 62 und die weiteren 61 AIT-Produkte, die sich im Entwicklungsprogramm der Therapieallergene-Verordnung (TAV) befinden, umfassen 95 Produkte zur subkutanen (SCIT) und 28 zur sublingualen Behandlung (SLIT) von Allergien gegen Birken‑/Erlen‑/Haselpollen, Gräser- und Kräuterpollen sowie Hausstaubmilben und Insektengifte. Als Wirkstoffe kommen native und chemisch modifizierte Allergenextrakte (Allergoide), adsorbiert an Aluminium, Tyrosin (z. T. Monophosphoryl-Lipid-A-adjuvantiert) oder Laktose, oder als Lyophilisate zum Einsatz.

Die 123 AIT-Produkte unterliegen der staatlichen Chargenprüfung. Individualrezepturen zur Behandlung weniger prävalenter Allergien (z. B. gegen Olivenpollen, Tierhaare, Speichermilben oder Schimmelpilze) unterliegen nicht der behördlichen Chargenprüfung. Besonderer Entwicklungsbedarf besteht für AIT-Produkte zur Behandlung von Kindern.

Als neue Wirkstoffklasse befinden sich Nahrungsmittelallergene in klinischen Phase-II- und Phase-III-Studien. Ein erstes Präparat zur oralen AIT der Erdnussallergie bei Kindern ist derzeit in einem zentralen europäischen Zulassungsverfahren. Die Zulassung kann nur bei positiver Nutzen-Risiko-Bilanz erfolgen. Wissenschaft und Regulation stehen in kontinuierlichem Austausch über die Entwicklung von AIT-Produkten, die dem aktuellen Stand der klinischen Forschung und Regulation in der Europäischen Union entsprechen und eine frühzeitige Kausalbehandlung weitverbreiteter Allergien ermöglichen.

## Abstract (en)

Allergen immunotherapy (AIT) is the only causally effective, disease-modifying form of therapy that, in addition to alleviating allergic symptoms, counteracts disease progression.

This article provides an up-to-date overview of immunological, regulatory and practical aspects of AIT. Current literature was included and recent conceptual regulatory developments from the Division of Allergology at the higher federal authority (Paul-Ehrlich-Institut) are presented.

The 62 AIT products currently approved in Germany and further 61 AIT products under the development program of the Therapy Allergen Ordinance (TAO) include 95 products for subcutaneous (SCIT) and 28 for sublingual (SLIT) treatment of birch/alder/hazel pollen, grass pollen, weed pollen, house dust mite and insect venom allergies. Native and chemically modified allergen extracts (allergoids) adsorbed to aluminium, tyrosine (partly monophosphoryl lipid A-adjuvanted) or lactose or based on lyophilisates are used as active ingredients.

These 123 AIT products are subject to official state batch release testing. This does not apply to named patient products (NPPs) available for the treatment of less prevalent allergies (e.g. to olive pollen, animal hair, storage mites or moulds). There is a particular need for development of AIT products for children.

As a new class of active ingredients, food allergens are in clinical phase II and III studies. A first food preparation for oral AIT of peanut allergy in children is currently undergoing a central European marketing authorization (MA) procedure. MA can only be granted if the benefit–risk balance is positive. Science and regulation are in continuous exchange on the development of AIT products that correspond to the current state of clinical research and regulation in the EU and enable early causal treatment of widespread allergies.

## Einleitung

Allergische Erkrankungen betreffen in Deutschland ca. 30 Mio. Menschen [1]. Es ist von über 3 Mio. ärztlich diagnostizierten Asthmaerkrankungen auszugehen, für Heuschnupfen liegt die Zahl der laut Selbsteinschätzung Betroffenen bei über 12 Mio. (>20 % der erwachsenen Bevölkerung) [2]. Allergische Erkrankungen verlaufen fast immer chronisch oder chronisch rezidivierend und schränken die Lebensqualität sowie die berufliche und schulische Leistungsfähigkeit der Betroffenen erheblich ein [1, 2]. Aktuelle Zahlen des Robert Koch-Instituts (RKI) und des Statistischen Bundesamtes (DESTATIS) [2–5] zur Verbreitung von Allergien bei Erwachsenen, Kindern und Jugendlichen sind in Infobox 1 zusammengefasst.

### Infobox 1 Epidemiologie von Allergien in Deutschland [2–5]

Nach aktuellen Zahlen des Robert Koch-Instituts (RKI) und des Statistischen Bundesamtes (DESTATIS) [2, 3] wird bei 30,0 % der 18- bis 79-jährigen Bevölkerung (36 % der Frauen und 24 % der Männer) in Deutschland mindestens eine allergische Erkrankung im Laufe des Lebens ärztlich diagnostiziert. Erfragt wurden dabei Asthma bronchiale, Heuschnupfen, Neurodermitis, Urtikaria (Nesselsucht), Kontaktekzeme, Nahrungsmittelallergien und Insektengiftallergien [2, 3]: Heuschnupfen und Asthma bronchiale waren bei Erwachsenen mit einer Lebenszeitprävalenz von 15,6 % bzw. 8,7 % am häufigsten; die dritthöchste Lebenszeitprävalenz war für das Kontaktekzem zu verzeichnen (8,6 %) [2]. Weniger prävalent waren jemals gestellte Arztdiagnosen für Nahrungsmittelallergie (5,0 %), Neurodermitis (3,7 %), Urtikaria (3,6 %) und Insektengiftallergie (3,0 %) [2]. Außer von Neurodermitis waren Frauen häufiger von allergischen Erkrankungen betroffen als Männer; besonders deutlich waren die Prävalenzunterschiede beim Kontaktekzem. Jüngere Erwachsene (bis 49 Jahre) sind häufiger von allergischen Erkrankungen betroffen als ältere [2, 3]. Die Häufigkeit allergischer Erkrankungen bei Erwachsenen bleibt in den letzten 10 Jahren auf hohem Niveau konstant, nur bei Asthma bronchiale gibt es Anzeichen für eine Zunahme [3].

Die Lebenszeitprävalenz von Kindern und Jugendlichen (im Alter von 0 bis 17 Jahren), an einer der 3 atopischen Erkrankungen (Asthma bronchiale, Heuschnupfen oder Neurodermitis) zu erkranken, lag bei 23 % in der Basiserhebung (2003–2006) der RKI-Studie zur Gesundheit von Kindern und Jugendlichen in Deutschland (KiGGS; [2]). Die Verbreitung von Heuschnupfen, Asthma und Neurodermitis bei Kindern und Jugendlichen in Deutschland blieb zwischen der KiGGS-Basiserhebung (2003–2006) und KiGGS Welle 2 (2014–2017) weitgehend konstant [4]. Als Lebenszeitprävalenzen von allergischen Erkrankungen (KiGGS Welle 2) wurden 6 % für Asthma bronchiale, 11 % für Heuschnupfen, 12,8 % für Neurodermitis und 2,8 % für allergisches Kontaktekzem festgestellt [4]. In der Querschnittsuntersuchung (2014–2017; KiGGS Welle 2) lag der Anteil der Kinder und Jugendlichen in der Altersgruppe der 11- bis 17-Jährigen mit ärztlicher Heuschnupfen- oder Neurodermitisdiagnose, die nach einem positiven Allergietest eine Allergenimmuntherapie erhielten, bei 30,1 % [5].

Für die allergenspezifische Immuntherapie (AIT) zur Kausalbehandlung von Allergien stehen in Deutschland und den Mitgliedsstaaten der Europäischen Union (EU) zugelassene oder verkehrsfähige Fertigarzneimittel für folgende Diagnosen (allergische Erkrankungen) zur Verfügung: Allergien gegen Inhalationsallergene (Rhinitis allergica, Conjunctivitis allergica, Asthma bronchiale) und Insektengiftallergien (Anaphylaxie) ([6]; Tab. 1). Einige Produkte verfügen auch über die Indikation „Prävention von allergischem Asthma bronchiale“ oder „Prävention von Neusensibilisierung gegen weitere Allergene“. Verfahren zur EU-weiten Zulassung erster AIT-Produkte zur Behandlung von Nahrungsmittelallergien wurden initiiert und sind derzeit bei den europäischen Zulassungsbehörden in Prüfung. Der vorliegende Beitrag gibt einen aktuellen Überblick zu a) immunologischer Wirkweise, b) Indikationen und Kontraindikationen der AIT, c) regulatorischen Voraussetzungen in Deutschland und der EU sowie d) neuen Wirkstoffen und Adjuvanzien.

**Table 1:** 

	Native Allergene							Modifizierte Allergene (Allergoide)							Anzahl der Produkte
	Birken‑, Erlen‑, Haselpollen	Gräserpollen und Mischungen aus Gräser‑/Getreidepollen (z. B. Roggen)	Birken‑, Erlen‑, Haselpollen/Gräserpollenmischungen	Hausstaubmilben	Kräuterpollen und Kräuterpollenmischungen (mit oder ohne Birken‑/Frühblüher- o. Gräserpollen)	Bienen‑, Wespengift	Weitere Allergenquellen	Birken‑, Erlen‑, Haselpollen	Gräserpollen und Mischungen aus Gräser‑/Getreidepollen (z. B. Roggen)	Birken‑, Erlen‑, Haselpollen/Gräserpollenmischungen	Hausstaubmilben	Kräuterpollen und Kräuterpollenmischungen (mit oder ohne Birken‑/Frühblüher- o. Gräserpollen)	Bienen‑, Wespengift	Weitere Allergenquellen	
**A) Zugelassene AIT-Produkte**															
*SCIT*															*53*
Wässrige Extrakte	–	–	–	–	–	–	–	–	–	–	–	–	–	–	0
Lyophilisiert	–	–	–	–	–	*n* = 8 (von 3 pU)	–	–	–	–	–	–	–	–	8
Aluminiumadsorbiert	*n* = 4 (von 1 pU)	*n* = 3 (von 1 pU)	–	*n* = 5 (von 2 pU)	–	*n* = 2 (von 1 pU)	–	*n* = 7 (von 2 pU)	*n* = 7 (von 2 pU)	*n* = 4 (von 2 pU)	*n* = 2 (von 1 pU)	*n* = 9 (von 1 pU)	–	–	43
Tyrosinadsorbiert	–	–	–	–	–	–	–	*n* = 1 (von 1 pU)	*n* = 1 (von 1 pU)	–	–	–	–	–	2
Tyrosinadsorbiert und MPL-adjuvantiert	–	–	–	–	–	–	–	–	–	–	–	–	–	–	0
*SLIT*															*9*
Tropfen	*n* = 4 (von 2 pU)	–	–	–	–	–	–	–	–	–	–	–	–	–	4
Tabletten	–	*n* = 2 (von 2 pU)	–	*n* = 1 (von 1 pU)	*n* = 1 (von 1 pU)	–	–	*n* = 1 (von 1 pU)	–	–	–	–	–	–	5
**B) Verkehrsfähige AIT-Produkte im Entwicklungsprogramm unter der Übergangsvorschrift der Therapieallergene-Verordnung (TAV)**															
*SCIT*															*42*
Wässrige Extrakte	–	–	–	–	–	–	–	–	–	–	–	–	–	–	0
Lyophilisiert	–	–	–	–	–	–	–	–	–	–	–	–	–	–	0
Aluminiumadsorbiert	–	–	–	–	–	–	–	*n* = 8 (von 2 pU)	*n* = 14 (von 2 pU)	*n* = 3 (von 2 pU)	*n* = 10 (von 3 pU)	–	–	–	35
Tyrosinadsorbiert	–	–	–	*n* = 1 (von 1 pU)	–	–	–	–	–	–	–	–	–	–	1
Tyrosinadsorbiert und MPL-adjuvantiert	–	–	–	–	–	–	–	2 (von 1 pU)	*n* = 1 (von 1 pU)	*n* = 2 (von 1 pU)	–	*n* = 1 (von 1 pU)	–	–	6
*SLIT*															*19*
Tropfen	*n* = 3 (von 2 pU)	*n* = 7 (von 3 pU)	–	*n* = 4 (von 3 pU)	–	–	–	*n* = 1 (von 1 pU)	*n* = 1 (von 1 pU)	–	–	–	–	–	16
Tabletten	–	–	–	–	–	–	–	*n* = 1 (von 1 pU)	*n* = 1 (von 1 pU)	–	*n* = 1 (von 1 pU)	–	–	–	3

*SCIT* subkutane Immuntherapie, *SLIT* sublinguale Immuntherapie, *n* Anzahl der Produkte, *MPL* Monophosphoryl-Lipid-A

## Immunologische Wirkweise der AIT

IgE-vermittelte Soforttypallergien wie Rhinokonjunktivitis allergica mit/ohne Asthma [7, 8], Insektengiftallergien [9] und Nahrungsmittelallergien [8] beruhen auf komplexen Gen-Umwelt-Interaktionen [10] und immunologisch vermittelter Überempfindlichkeit (Tab. 2). Nach initialer Sensibilisierung gegen definierte (Glyko)Proteine entsteht eine allergenspezifische T‑Helfer-Typ-2-(Th2-)Immunantwort. Bei erneutem Allergenkontakt aggregieren neu gebildetes IgE und seine Rezeptoren auf Mastzellen und basophilen Granulozyten, deren Granulation die (u. a. histaminvermittelte) Sofort- oder Frühreaktion auslöst. Anschließend infiltrieren weitere Leukozyten das Gewebe und setzen weitere Mediatoren frei (Spätphase der allergischen Entzündung). Sowohl allergische Überreaktion (Tab. 2) als auch AIT (Tab. 3) beruhen auf dem Zusammenwirken diverser Zellen und Mediatoren. Tab. 2 und Tab. 3 geben einen aktuellen Überblick über den derzeitigen Kenntnisstand zu den wichtigsten wirksamen immunologischen Mechanismen.

**Table 2:** 

Zellen/Mediatoren	Effekte
Allergenspezifisches IgE	Nach z. B. nasaler Allergenexposition erfolgt eine IgE-abhängige Aktivierung von Mastzellen und Basophilen mit unmittelbarer Soforttypreaktion (Eintritt nach 0 bis 60 Minuten). Zusätzlich zu den systemischen und regionalen lymphatischen IgE-Quellen kann spezifisches IgE lokal von B‑Zellen innerhalb des Atemwegstraktes produziert werden; dies erklärt für eine sog. lokale allergische Rhinitis mit Symptomen bei Allergenexposition in Abwesenheit von nachweisbarem allergenspezifischen Serum-IgE oder positiven Hauttestergebnissen gegen relevante Allergene
Mastzellen, basophile Granulozyten	Nach vorangegangener Sensibilisierung vernetzt bei Zweitkontakt das Allergen benachbarte Oberflächen-IgE-Moleküle auf Mastzellen und Basophilen innerhalb von Sekunden oder Minuten, die anschließend präformierte, intrazelluläre Mediatoren wie Histamin und Tryptase freisetzen. Zu den neu gebildeten Lipidmediatoren gehören die Sulfidopeptid-Leukotriene (LTs; LTC4, LTD4 und der terminale Metabolit LTE4), der thrombozytenaktivierende Faktor (PAF) und Prostaglandin D2 Stunden nach der Allergenexposition synthetisieren und setzen Mastzellen auch proinflammatorische Zytokine (z. B. IL‑6, Tumornekrose Faktor α (TNFα)), Th2-Zytokine (z. B. IL‑4, IL‑5, IL-13) und Chemokine (z. B. CCL3) frei, vermittelt durch mehrere Transkriptionsfaktoren (einschließlich „nuclear factor ‚kappa-light-chain-enhancer‘ of activated B‑cells“ (NF-κB), einem zentralen Faktor bei der Regulation von Immunantwort, Entzündungsreaktion und Zellüberleben); die Expression von IL‑6 in aktivierten Mastzellen wird vermittelt durch NF-κB, während die Expression von TNFα und IL-13 in aktivierten Mastzellen „nuclear factor of activated T cells“ (NFAT) erfordert
Histamin	Histamin entfaltet seine Wirkungen durch Bindung an membrangebundenen Histaminrezeptoren (H_1_-, H_2_-, H_3_- und H_4_-Rezeptoren). Unmittelbare allergische Symptome (Vasodilatation, Erhöhung der Gefäßpermeabilität und Bronchokonstriktion) sind überwiegend durch die Histaminwirkung an Histamin(H)1-Rezeptoren der Zielorgane vermittelt
Leukotriene, PAF, Prostaglandin D2	Die biologischen Eigenschaften dieser Mediatoren fördern die lokale Vasodilatation, Ödembildung, lokale neurogene Stimulation und Schleimsekretion, die die typische nasale allergeninduzierte unmittelbare Typ-I-Überempfindlichkeit charakterisieren. Sie tragen zur Aufrechterhaltung der Entzündungsreaktion durch Anlockung von Neutrophilen und Eosinophilen bei. In den unteren Atemwegen tragen sie zur Kontraktion der glatten Bronchialmuskulatur sowie über die Ödembildung und Schleimhypersekretion zur akuten Bronchokonstriktion bei
Eosinophile Granulozyten	Eosinophile werden in der Atemwegsschleimhaut als Reaktion auf das von Th2-Zellen und innate lymphoide Zellen (ILC) produzierte IL‑5 rekrutiert. Die späte Reaktion, die 4–12 h nach Allergenexposition durch Gewebeeosinophilie, nasale Kongestion und mukosale Hyperreaktivität auf allergische und nichtallergische Auslöser gekennzeichnet ist, kann nach einer einzigen nasalen Allergenexposition Tage oder sogar Wochen andauern. Eosinophile sind in der allergischen Kaskade essenziell und sie produzieren reaktive Sauerstoffspezies wie Wasserstoffperoxid und Superoxidanion, die zu Epithelschädigung, intensiver Entzündungsreaktion und Aktivierung verschiedener Signalkaskaden führen
Respiratorische Epithelzellen	Gelangen bei entsprechender atopischer Anlage Aeroallergene durch das entzündete Nasenepithel, setzen aktivierte Epithelzellen die Chemokine CCL2 und CCL20 frei, die unreife dendritische Zellen (DCs) rekrutieren Das respiratorische Epithel von Individuen mit allergischer Disposition exprimiert Zytokine, zu denen IL-25, IL-33 und das thymische stromale Lymphopoietinprotein (TSLP) gehören. Diese epithelialen Zytokine begünstigen die Entwicklung eines proallergischen Phänotyps an dendritischen Zellen, der eine TH2-Zelldifferenzierung unterstützt. Darüber hinaus sind diese epithelial abgeleiteten Zytokine wichtige Wachstumsfaktoren für Lymphozyten des angeborenen Immunsystems (innate lymphoide Zellen der Gruppe 2 (ILC2s)), die die lokale TH2-getriebene allergische Entzündung verstärken und aufrechterhalten
Airway Remodeling	Im Gegensatz zu Befunden bei Patienten mit allergischem Asthma und Nasenpolyposis gehören selbst bei mittelschwerer/schwerer allergischer Rhinitis morphologische und immunhistochemische Merkmale von Umbauvorgängen der Atemwege nicht zum Krankheitsbild
Dendritische Zellen (DC)	Die DC-Migration wird durch IL-13, das von ILC2s produziert wird, und auch durch IL‑4, das hauptsächlich von Basophilen produziert wird, ausgelöst. Aktivierte DCs wandern zu regionalen drainierenden Lymphknoten und polarisieren naive T‑Zellen zu TH2-Zellen. Während der anschließenden Allergenexposition verstärkt die IgE-unterstützte Allergenerkennung durch den hochaffinen Rezeptor FcεRI auf DCs und FcεRII auf B‑Zellen die Entwicklung von TH2-Reaktionen auf Inhalationsallergene DCs können in Abhängigkeit von ihrer Reifungsphase, ihrer Lokalisation und dem damit verbundenen lokalen Zytokinmilieu entweder eine allergische Entzündung auslösen und aufrechterhalten (proallergische DC2s) oder alternativ einen Zustand der Immuntoleranz (tolerogene regulatorische dendritische Zellen (DCregs)) gegenüber Allergenen fördern. DC2s exprimieren die Marker CD141, GATA‑3, OX40-Ligand und die rezeptorinteragierende Serin/Threonin-Proteinkinase 4 (RIPK4)
TH2-Lymphozyten	TH2-Zellen produzieren IL‑4, das Schlüsselzytokin, das für die TH2-Zelldifferenzierung verantwortlich ist. Die Freisetzung der TH2-Zytokine IL‑4, IL‑5, IL‑9, IL-13 und die Gewebeeosinophilie zeigen sich während der Spätphasenreaktion, die 4–12 h nach der Allergenexposition auftritt Die TH2-Zelldifferenzierung ist abhängig vom lokalen Zytokinmilieu, das bestimmt wird durch Interaktionen zwischen dem respiratorischen Epithel, lokalen dendritischen Zellen und regionalen Lymphknoten
Follikuläre Helfer-T-Zellen (TFH)	Innerhalb des Keimzentrums des Lymphknotens differenziert eine Untergruppe von TH-Zellen in follikuläre Helfer-T-Zellen (TFH). TFH-Zellen produzieren sowohl IL‑4 als auch IL-21, die zusammen mit dem von TH2-Zellen stammenden IL‑4 den Immunglobulin-Schwere-Kette-Klassenwechsel zu IgE in B‑Zellen fördern
B‑Lymphozyten	IL‑4 und IL-21 induzieren die Produktion von allergenspezifischem IgE durch B‑Zellen. Während des Isotyp-Switch im Keimzentrum der Lymphknoten werden bestimmte B‑Zelluntergruppen zu Plasmazellen, die von der IgM- zur IgE-Produktion wechseln; diese IgE-Antikörper binden schließlich an den hochaffinen Rezeptor (FcεRI) auf der Oberfläche von Mastzellen und Basophilen, was zur Sensibilisierung führt. IgE kann auch von B‑Zellen innerhalb der Atemwegsschleimhaut synthetisiert und lokal produziert werden. Während der anschließenden Allergenexposition verstärkt die IgE-unterstützte Allergenerkennung durch FcεRI auf DCs und FcεRII auf B‑Zellen die Entwicklung von TH2-Reaktionen auf Inhalationsallergene
IL‑4 und IL-13	IL‑4 und IL-13 induzieren B‑Lymphozyten zur Produktion von ε‑germline-Gentranskripten, dem ersten Schritt des Gen-Rearrangements der schweren Kette zugunsten der IgE-Produktion. IL‑4 und IL-13 regulieren die Expression des vaskulären Zelladhäsionsproteins 1 (VCAM 1) auf dem Gefäßendothel hoch, wodurch die Adhäsion von das Very Late Antigen (VLA) 4 (=Integrin a4β1) exprimierenden Eosinophilen gefördert wird. Beide stimulieren die Schleimproduktion der Drüsen in den oberen und unteren Atemwegen
IL‑5	IL‑5 ist für die terminale Differenzierung und Freisetzung von Eosinophilen aus dem Knochenmark verantwortlich und verlängert das Überleben der Eosinophilen, indem es die Apoptose der Eosinophilen in den Geweben hemmt
Innate lymphoide Zellen (ILCs)	ILCs bestehen aus 3 verschiedenen Gruppen, die als Gruppe-1-ILCs, Gruppe-2-ILCs und Gruppe-3-ILCs bezeichnet werden. ILCs der Gruppe 1 exprimieren konstitutiv den T‑Box-Transkriptionsfaktor und produzieren die TH1-Zytokine Interferon-(IFN-)γ und TNF und bieten Schutz gegen intrazelluläre Bakterien und Parasiten. ILC2s exprimieren konstitutiv den Transkriptionsfaktor RAR-related-Orphan-Rezeptor (ROR) α und GATA‑3; sie produzieren TH2-Zytokine, insbesondere IL‑5 und IL-13, und bieten Immunität gegen Helminthen und stimulieren allergische Reaktionen. ILC der Gruppe 3 sind durch den Transkriptionsfaktor RAR-related-Orphan-Rezeptor (ROR) γt charakterisiert, exprimieren IL-17a und/oder IL-22, bieten Schutz gegen extrazelluläre Bakterien und sind an Gewebereparaturprozessen beteiligt
Gruppe-2-innate lymphoide Zellen (ILC2s)	ILC2s hängen ebenfalls von o. g. epithelialen Zytokinen als Wachstumsfaktoren ab und stellen eine alternative Quelle von TH2-Zytokinen in der Nasenschleimhaut dar. Angeborene Lymphoidzellen (ILCs) sind den Lymphozyten morphologisch ähnlich, obwohl sie sich dadurch unterscheiden, dass sie keine Oberflächenantigenrezeptoren oder andere Zelllinienmarker exprimieren und antigenunabhängig agieren. Die Rolle der ILC2s bei allergischer Rhinitis wurde erstmals bei Patienten mit Katzenallergie identifiziert, die 4 h nach einer nasalen Provokation des Katzenallergens einen Anstieg der ILC2-Zahlen im peripheren Blut zeigten. In der Folge wurden Erhöhungen der zirkulierenden ILC2-Zahlen sowohl bei Patienten mit Gräserallergie und Rhinitis als auch bei Asthmatikern während der Gräserpollensaison festgestellt ILC2s stellen eine reichlich vorhandene alternative Quelle von TH2-Zytokinen dar und dienen wahrscheinlich dazu, lokale TH2-getriebene allergische Entzündungen zu verstärken und aufrechtzuerhalten

**Table 3:** 

Zellen/Mediatoren	Effekte
Allergenspezifisches IgE	Sowohl die sublinguale Immuntherapie (SLIT) als auch die subkutane Immuntherapie (SCIT) sind mit vorübergehenden frühen Anstiegen der serumallergenspezifischen IgE-Antikörperspiegel assoziiert, auf die eine Abflachung der üblichen saisonalen Anstiege der IgE-Spiegel während der natürlichen Allergenexposition folgt. Diese frühen Anstiege werden nicht von unerwünschten Nebenwirkungen begleitet und es wird angenommen, dass ein frühes TH2-Priming durch hohe Allergenexposition für eine erfolgreiche Immuntherapie wichtig sein könnte. Längere SCIT über mehrere Jahre kann zu einer Abnahme der allergenspezifischen IgE-Konzentrationen (sIgE) führen, ein Ereignis, das zur Langzeittoleranz beitragen könnte
IgG, IgG4, and IgA („blocking antibodies“)	Es wurde gezeigt, dass die inhibitorischen Effekte für IgE nasal und im Serum nach SCIT innerhalb der Serum-IgG-, IgG4- und IgA-Fraktionen liegt. Studien haben gezeigt, dass die Serumkonzentrationen von IgG, insbesondere IgG4, um das 10- bis 100-fache ansteigen. Auch SLIT induziert allergenspezifische IgG1-, IgG4- und IgA-Antikörper. Diese Zunahme immunreaktiver Antikörper wurde nach Immuntherapie gegen saisonale Pollen als auch gegen perenniale Allergenquellen, wie z. B. Hausstaubmilben, beobachtet. Mehrere Studien haben die hemmende Kapazität von IgG4 für IgE-abhängige Reaktionen hervorgehoben. IgG4-Antikörper sind bispezifisch und haben die Fähigkeit zum Austausch von F(ab)-Armen durch den Austausch von schwer-leichten Kettenpaaren zwischen IgG4-Molekülen mit unterschiedlichen Spezifitäten. Obwohl die immunreaktiven IgG/IgG4-Spiegel innerhalb eines Jahres nach Absetzen der Allergenimmuntherapie um 80–90 % sanken, blieb die IgG-assoziierte serum-IgE-hemmende Aktivität mehrere Jahre lang bestehen und ging mit einer langfristigen klinischen Wirksamkeit einher. Dies deutet darauf hin, dass IgG-Antikörper, die nach Absetzen der Immuntherapie persistieren, trotz niedrigerer Spiegel entweder eine höhere Avidität oder eine höhere Affinität aufweisen. IgG-Antikörper wurden auch lokal nach der Immuntherapie sowohl in der Nasenflüssigkeit als auch im Serum nachgewiesen. Sowohl die spezifischen IgG4-Spiegel als auch die assoziierte inhibitorische Aktivität für die IgE-vermittelte Antigenbindung (IgE-FAB) waren in der Nasenflüssigkeit von Patienten, die sich einer SLIT unterzogen, im Vergleich zu unbehandelten Teilnehmern erhöht. Die IgG4-Abhängigkeit der IgE-inhibierenden Aktivität wurde in Depletionsexperimenten mittels IgG4-Affinitätschromatographie gezeigt. Das Ausmaß der IgE-Suppression war mit Nasenflüssigkeit größer als mit Serum, was die Potenz der lokalen IgG-inhibierenden Antikörper verdeutlicht
Mastzellen, basophile Granulozyten	Die signifikante Abnahme der Anzahl der Effektorzellen, einschließlich CD117+(c‑kit+)-Mastzellen, Basophilen und Eosinophilen in der Nasenschleimhaut im Vergleich zum Ausgangsbefund vor Behandlung, konnte in einer doppelblinden Gräserpollen-SCIT-Studie im Vergleich zur Placebogruppe gezeigt werden, was mit einer klinischen Verbesserung der Symptomatik und einem geringeren Verbrauch an Bedarfsmedikation einherging
Histamin	IgG kann mit IgE um das Allergen konkurrieren und dadurch die Bildung des Allergen-IgE-Komplexes blockieren. Dadurch wird die Quervernetzung von hochaffinen IgE-Rezeptoren (FcεRI) auf Basophilen und Mastzellen verhindert und die Histaminfreisetzung gehemmt Sowohl SCIT als auch die SLIT hemmen Früh- und Spätphasereaktionen nach einer Allergenprovokation. Die Suppression geht mit einer Verringerung des frühen Anstiegs von lokalem nasalen Histamin, Tosyl-L-Arginin-Methylesterase und Tryptasekonzentration in der Nasenflüssigkeit einher
Eosinophile Granulozyten	Die Hemmung der Spätreaktionen nach AIT ist mit einer Abnahme der Anzahl der Eosinophilen und der Konzentrationen der TH2-Zytokine, einschließlich IL‑4, IL‑5, IL‑9 und IL-13, verbunden. Adhäsions- und chemotaktische Faktoren für Eosinophile nehmen nach der AIT ab, einhergehend mit einer verminderten bronchialen Hyperreaktivität
Respiratorische Epithelzellen	Zum AIT-Einfluss auf die innate Zytokineantwort von Epithelzellen, die an der Regulation sowohl der lokalen TH2-Reaktion als auch von ILC beteiligt sind, stehen Forschungsergebnisse aus
Dendritische Zellen (DC)	Die Wangenschleimhaut ist ständig Fremdproteinen in Lebensmitteln ausgesetzt und stellt ein ausgeprägtes protolerogenes Milieu dar. Ex-vivo-Studien von Wangenschleimhautbiopsieproben von Patienten mit Gräserpollenallergie haben gezeigt, dass Langerhans-Zellen der Mundschleimhaut das Hauptgräserpollenallergen „Phl p 5“ dosis- und zeitabhängig binden. Nach 5 min tritt ein Plateau ein, was zu einer verlangsamten Reifung der oralen Langerhans-Zellen führt, parallel zu einer verbesserten Migrationsfähigkeit und einer erhöhten Produktion von tolerogenen Zytokinen, die IL-10 und Transforming Growth Factor (TGF) β umfassen Es wurde der AIT-Einfluss auf Subtypen von DCs im Blutkreislauf untersucht. PCR-Studien von peripheren Blutproben, die vor und nach einer 4‑monatigen sublingualen Gräserpollenimmuntherapie entnommen wurden, wurden zur Charakterisierung von Veränderungen des DC-Phänotyps verwendet: Eine signifikante Zunahme der Zahl der DCs des DCreg-Phänotyps wurde beobachtet. Die DCreg-Signatur spiegelte sich in einer Zunahme der mRNA-Expression für Stabilin‑1 und die Komplementkomponente 1Q (C1Q) wider. Interessanterweise wurde diese DCreg-Signatur nur bei denjenigen beobachtet, die auf die Immuntherapie ansprachen, was sich in einer signifikanten Abnahme der Rhinokonjunktivitissymptome widerspiegelte. Zur Untermauerung dieser Befunde führte eine einjährige SLIT-Behandlung von Kindern mit Milbenallergie zu peripheren DCs, die einen unreifen Phänotyp und eine erhöhte Fähigkeit zur Produktion von IL-10 und verringerte IL-12-Spiegel aufwiesen
TH2-Lymphozyten	Die Suppression von allergeninduzierten späten nasalen Reaktionen während einer Gräserpollen-SCIT war mit einer Abnahme der Anzahl von CD4+-T-Zellen und lokalen IL‑4 mRNA-positiven T‑Zellen in der Nasenschleimhaut assoziiert. Diese Befunde werden durch die Beobachtung von Abnahmen der TH2-Zytokinspiegel in der Nasenschleimhautflüssigkeit nach nasaler Provokation gestützt In einer Studie zum Ansprechen der allergischen Rhinitis auf SCIT versus SLIT (GRASS) führte sowohl die subkutane als auch die sublinguale Immuntherapie während 2 Jahren zu einer klinischen Verbesserung, die mit einer Abnahme der Anzahl peripherer tetramer-positiver CRTH2+CCR4+CD27-CD4+-TH2-Zellen einherging. Parallel zu diesen Veränderungen kam es zu einer Reduktion der Spiegel lokaler nasaler TH2-Zytokine, einschließlich IL‑4, IL‑5 und IL-13, in der Nasenflüssigkeit nach nasaler Allergenprovokation. Sowohl die Anzahl zirkulierender tetramer-positiver TH2-Zellen als auch die Konzentrationen lokaler nasaler TH2-Zytokine stiegen im Laufe des dritten Jahres wieder an, zusammen mit einer Verschlechterung der saisonalen Symptome ein Jahr nach Absetzen der Immuntherapie
TH1-Immundeviation	Die Suppression der TH2-Immunreaktion bei SCIT und SLIT geht auch mit einer Immundeviation und Induktion von TH1-Zellen einher. In-situ-Hybridisierungsstudien der Nasenschleimhaut nach erfolgreicher SCIT zeigten die Zunahme der IFNG-mRNA+-T-Zellen nach Allergenprovokation, die mit einer Abnahme der nasalen Symptome während der Pollensaison korrelierte. Pollen-AIT war mit einer Abnahme des Verhältnisses von IL5/IFNG-mRNA+-Zellen in der Schleimhaut und einem Anstieg der nasalen IFNγ-Proteinspiegel in der Nasenflüssigkeit während der natürlichen saisonalen Allergenexposition assoziiert. In ähnlicher Weise führte die subkutane Gräserpollenimmuntherapie zu einem Anstieg der IL12-mRNA+-Makrophagen in der Haut, der mit einer Suppression der späten Hautreaktionen einherging und positiv korrelierte mit lokalen IFN-γ+-T-Zellen und invers mit IL-4-exprimierenden T‑Zellen. Der Nachweis für/gegen eine TH1-Deviation im peripheren Blut ist in Studien kontrovers. Eine Studie deutete darauf hin, dass die Verschiebung von TH2- zu TH1-Reaktionen mit dem Zelltod der allergenaktivierten TH2-Zellen in Verbindung stehen könnte. Darüber hinaus wurde über das Überleben von TH1-Zellen nach der Deletion von TH2-Zellen berichtet
Regulatorische T‑Zellen (Treg)	Immuntoleranz während der AIT geht mit der Induktion von allergenspezifischen regulatorischen CD4+-T-Zellen (Treg) einher. Treg-Zellen können in 2 Untergruppen eingeteilt werden: natürliche regulatorische T-(nTreg‑)Zellen, die den Transkriptionsfaktor Forkhead Box P3 (FOXP3) exprimieren, und induzierbare regulatorische T-(iTreg‑)Zellen, die regulatorische Zytokine wie IL-10, IL-35 und TGF‑β produzieren. nTreg-Zellen haben zusätzlich zum Transkriptionsfaktor FOXP3 eine erhöhte Expression des IL-2-Rezeptors (CD25) und eine niedrige Expression des IL-7-Rezeptors (CD127). nTreg-Zellen üben ihre Suppressionsfähigkeit in einer direkten zell-zell-kontakt-abhängigen Weise aus. Funktionelle Bedeutung wurde dem zytotoxischen T‑lymphozytenassoziierten Protein 4 (CTLA-4), dem oberflächengebundenen TGF‑β, dem glukokortikoidinduzierten TNF-Rezeptor und PD‑1 („programmed cell death protein 1“) zugeschrieben. nTreg-Zellen modulieren auch allergenspezifische T‑Zellantworten gesunder nichtatopischer Personen. SCIT war assoziiert mit lokalem Anstieg der FOXP3+CD25+-T-Zellzahlen in der Nasenschleimhaut im Vergleich zu unbehandelten Kontrollpersonen Immunfluoreszenzuntersuchungen von sublingualen Biopsien identifizierten einen Anstieg der FOXP3+CD3+-Zellzahlen in der sublingualen Schleimhaut nach Gräserpollen-SLIT. In-vitro-Untersuchungen von Biopsien menschlicher Wangenschleimhaut und zugehöriger Zungenmandeln identifizierten die Oropharynxschleimhaut als reich an protolerogenen DCs und Treg-Zellen. Eine veränderte nTreg-Zellfunktion wurde mit epigenetischen Modifikationen bei der FOXP3-Promotorregion in Verbindung gebracht
	Follikulläre Helfer T-(TFH)-Zellen sind durch Oberflächen-CXCR5, das Transkriptionsfaktor-B-Zell-Lymphom-6-Protein, und eine erhöhte Expression von IL‑4, IL-21 und IL‑6 gekennzeichnet. TFH-Zellen befinden sich in den Randzonen von Keimfollikeln innerhalb regionaler Lymphknoten, wo sie eine wesentliche Hilfe für die B‑Zellreifung und den Klassenwechsel darstellen. Im Jahr 2004 wurde eine bestimmte Population von CXCR5-exprimierenden FOXP3+-Treg-Zellen identifiziert, die die Fähigkeit besitzen, in Keimzentren einzuwandern und T‑ und B‑Zellreaktionen zu supprimieren. Diese Zellpopulation wurde erst 2011 als eine eigenständige Untergruppe von CD4+-T-Zellen mit regulatorischer Kapazität als follikuläre regulatorische T‑Zellen (TFR-Zellen) anerkannt. Eine Studie zeigte, dass die TFH-Gedächtniszellen nach einer Immuntherapie signifikant reduziert waren Darüber hinaus wurde gezeigt, dass TFR-Zellen von immuntherapeutisch behandelten Patienten eine vergleichsweise höhere Produktion von IL-10 aufweisen. Wenn CXCR5+-TFH-Zellen von immuntherapeutisch behandelten Spendern angereichert und in Gegenwart von T‑Zellrezeptorstimulation und IL‑2 für 5 Tage kultiviert werden, zeigt die durchflusszytometrische Analyse einen Anstieg der TFR-Zellzahlen. Diese Ergebnisse unterstreichen die Plastizität der TFR-Zellen und ihre wahrscheinliche Rolle bei der Suppression von TH2-Antworten und der Produktion von IgE-Antikörpern während der Allergenimmuntherapie
	iTreg-Zellen produzieren entweder IL-10 (TR1) oder TGF‑β (TH3) und modulieren die allergengesteuerte proliferative Reaktion von T‑Zellen und die Freisetzung von TH2-Zytokinen. Studien an nasalen Biopsien, die vor und 2 Jahre nach der Gräserpollenimmuntherapie gewonnen wurden, ergaben eine Verschiebung zugunsten lokaler iTreg-Zellantworten in der Nasenschleimhaut. Während der Pollensaison wurde eine Zunahme der Anzahl IL-10-exprimierender T‑Zellen beobachtet, die mit einem Anstieg der Serum-IgG4-Spiegel verbunden war. Ein saisonaler Anstieg der TGF-β+-T-Zellen in der Nasenschleimhaut korrelierte mit einem Anstieg der peripheren zirkulierenden IgA-Konzentration. Nach SLIT mit Gräser- und Birkenpollen wurde über die Induktion peripherer IL-10+-Treg-Zellen berichtet. Während einer Gräserpollen-SCIT zeigte sich im Zeitverlauf, dass bereits mononukleäre Zellen des peripheren Blutes (PBMCs), die nach 2 bis 4 Wochen während der frühen Dosissteigerung gewonnen wurden, nach 6‑tägiger Co-Kultivierung mit Gräserpollenallergen hohe IL-10-Spiegel in den Überständen produzierten. Dieses frühe IL-10-Signal war eng mit der Suppression der allergeninduzierten Late-Phase-Reaktion verbunden. Auf Erhöhungen der IL-10-Produktion und die Unterdrückung der Spätreaktion folgten nacheinander Erhöhungen der Serum-IgG4-Spiegel nach 6 bis 8 Wochen, die ihren Höhepunkt nach 16 Wochen erreichten, zusammen mit einer parallelen Suppression der frühen Hautreaktionen. Es zeigte sich, dass das Serum nach der Immuntherapie eine IgG-assoziierte IgE-Blockierungsaktivität sowohl für die Basophilenaktivierung (erhöhte allergenstimulierte Basophilen-CD63) als auch für die IgE-FAB-Inhibition aufwies, die parallel zu den Anstiegen der IgG4-Spiegel verlief iTreg-Zellen produzieren auch IL-35, ein neu identifiziertes inhibitorisches Zytokin aus der IL-12-Familie heterodimerer Zytokine, die eine entzündungshemmende Reaktion hervorrufen. Das von iTregs produzierte IL-35 unterdrückt die durch ILC2s und Th2-Zellen vermittelte Typ-2-Immunantwort bei Patienten mit allergischer Rhinitis unter Gräserpollen-SLIT
B‑Lymphozyten	Die Kompetition von IgG/IgG4 mit IgE kann auch die Bindung von Allergen-IgE-Komplexen an Rezeptoren mit niedriger Affinität (FcγRIIb) auf B‑Zellen blockieren und dadurch die IgE-unterstützte Antigenpräsentation gegenüber T‑Zellen hemmen, die ein Hauptfaktor für allergenspezifische TH2-Reaktionen ist. Jüngste Daten weisen auf die Möglichkeit hin, dass durch Immuntherapie induzierte langlebige Gedächtnis-B-Zellen infolge einer schwachen Stimulation durch Umweltallergene persistieren und dadurch zur Langzeittoleranz beitragen können
Regulatorische B‑Zellen (Breg)	Regulatorische B‑Zellen (Breg) sind eine Untergruppe von B‑Zellen, die IL-10 produzieren und die Fähigkeit besitzen, T‑zell- und DC-vermittelte Entzündungsreaktionen zu hemmen und die natürliche Immuntoleranz aufrechtzuerhalten. Gereinigte Populationen IL-10-produzierender Breg-Zellen von bienengifttoleranten Probanden wiesen eine hohe Oberflächenexpression von CD25 und CD71 und eine Expression von CD73 auf. Darüber hinaus konnte gezeigt werden, dass die allergenspezifischen IgG4-Antikörper nach einer Bienengiftimmuntherapie von phospholipase-A2-spezifischen IL-10+Breg-Zellen stammten. Die suppressiven Eigenschaften regulatorischer B‑Zellen werden durch ihre Produktion von TGF‑β und IL-35 vermittelt. Ob der gleiche Phänotyp von B‑Zellen nach einer Immuntherapie mit Inhalationsallergenen exprimiert wird, ist noch zu klären. Kürzlich wurde zum ersten Mal ein Anstieg der Zahl der IL-10+-Breg-Zellen unter Gräserpollen-SCIT gezeigt, der mit dem Anstieg des allergenspezifischen IgG4-Spiegels in der Nasenflüssigkeit einherging; die durch spezifische IgG4-Antikörper verursachte hemmende Aktivität korrelierte eng mit der klinischen Reaktion auf die SCIT
IL‑4 und IL-13	Die Hemmung der Spätreaktionen nach AIT geht mit einer Abnahme der Konzentrationen der TH2-Zytokine, einschließlich IL‑4, IL‑5, IL‑9 und IL-13, einher
IL‑5	Es wurde eine direkte Korrelation zwischen der Reduktion der IL-5-Spiegel und der Anzahl nasaler mukosaler Eosinophilen und auch zwischen Eosinophilenzahlen und dem Schweregrad der saisonalen Symptome festgestellt
Gruppe-2-innate lymphoide Zellen (ILC2s)	Der Einfluss der Immuntherapie auf ILC2-Zellen wurde im peripheren Blut, aber nicht in den Zielorganen untersucht, zum Teil wegen der Schwierigkeiten bei der Identifizierung dieser Zellen, die keine Zelllinienmarker exprimieren, die einer immunhistochemischen Lokalisierung in Geweben zugänglich sind. Nach einer subkutanen Gräserpollenimmuntherapie kam es zu einer deutlichen Hemmung der saisonalen Zunahmen der liniennegativen CRTH2+CD127+-ILC2s, die mit der Schwere der selbstberichteten Symptome während der Pollensaison korrelierten. Diese Ergebnisse waren hochsignifikant im Vergleich zu den saisonalen Zunahmen der ILC2-Zahlen, die bei gematchten unbehandelten Kontrollpersonen mit saisonaler allergischer Rhinitis beobachtet wurden. Diese Daten wurden durch die Hemmung des saisonalen Anstiegs der Zahl der CD117+-(c-kit1-)ILC2 und des Anteils der IL-13+-ILC2, der mittels intrazellulärer Zytokinfärbung bestimmt wurde, unterstützt. In einer Studie zur Immuntherapie bei Teilnehmern mit saisonalem Asthma gab es keine Veränderung in der Anzahl der ILC2s, obwohl dies wahrscheinlich dadurch erklärt werden kann, dass die Messungen außerhalb der Saison durchgeführt wurden, als die Teilnehmer asymptomatisch waren

Bei einer AIT wird eine Immunmodulation durch verschiedene Mechanismen (Tab. 3) erzielt: Der Einfluss der AIT auf Effektorzellen wurde besonders nach nasaler Allergenprovokation und während saisonaler Pollenbelastung untersucht [11]. Sowohl die subkutane (SCIT) als auch die sublinguale Immuntherapie (SLIT) hemmen frühe und späte Reaktionen nach einer Allergenexposition. Die hochdosierte Allergenzufuhr bei der AIT stellt die Funktion allergenpräsentierender dendritischer Zellen und deren Interleukin-(IL-)12-, IL-27- und IL-10-Produktion wieder her. Das fördert eine Immundeviation von einer Th2- zur Th1-Reaktion und aktiviert regulatorische Immunzellen (Treg, Breg) und andere B‑Zellen, die allergenspezifische blockierende Immunglobulin-(Ig‑)A-, IgG- und IgG4-Antikörper produzieren (Tab. 3).

Trotz intensiver Suche gibt es bisher keinen Biomarker, der zu Beginn oder nach Abschluss der AIT eine verlässliche Aussage zum Ansprechen auf die Behandlung zulässt [12, 13].

## Indikationen/Kontraindikationen von AIT-Produkten

AIT ist die einzige krankheitsmodifizierende und kurative Therapieform bei der Behandlung von Soforttypallergien (Typ-I-Allergie). Im Gegensatz zu symptomatischen pharmakologischen Therapien verfolgt die AIT das Ziel einer anhaltenden immunologischen Toleranz gegen die Allergieauslöser.

Die klinische Indikation besteht bei nachgewiesener IgE-vermittelter Sensibilisierung mit zughörigen klinischen Symptomen, sofern geeignete AIT-Produkte zur Verfügung stehen. Dabei werden Vorerkrankungen, Medikation und zusätzliche (relative) Kontraindikationen berücksichtigt, die in klinischen Leitlinien der wissenschaftlichen Fachgesellschaften erläutert werden [11, 14–19]. Im Folgenden werden aktuelle produktseitige Aspekte zu ausgelobten Indikationen/Kontraindikationen thematisiert.

### Klinische Studien als Basis der produktspezifischen Indikationen

Die jeweilige Indikation, für die ein AIT-Produkt zugelassen wird, muss durch mindestens zwei klinische Phase-III-Studien in ihrer Wirksamkeit und Sicherheit mit einem positiven Nutzen-Risiko-Verhältnis belegt sein. Bei herausragender Ergebnisqualität kann in Ausnahmefällen eine klinische Phase-III-Studie ausreichend sein [20]. Die produktspezifischen Indikationen und Kontraindikationen sind der jeweiligen Fachinformation (unter 4.1 Anwendungsgebiete und unter 4.3 Gegenanzeigen) zu entnehmen. Welche Indikationen geltend gemacht werden können, basiert auf der erbrachten klinischen Datenlage in Abhängigkeit von Studiendesign/-dauer ([21]; Tab. 4).

**Table 4:** 

Indikation	Studiendesign/-dauer
„Behandlung allergischer Symptome“	Nachweis der Wirksamkeit in klinischen Kurzzeitstudien in der ersten Pollensaison nach AIT-Beginn oder bei ganzjährig auftretenden Allergien nach einigen Monaten der Behandlung
„Anhaltende klinische Wirkung“	Aufrechterhaltung der signifikanten und klinisch relevanten Wirksamkeit während 2–3 Behandlungsjahren
„Langfristige Wirksamkeit und krankheitsmodifizierende Wirkung“	Anhaltend signifikante und klinisch relevante Wirksamkeit 2 Jahre nach der Behandlung
„Heilung von Allergien“	Anhaltende Abwesenheit allergischer Symptome in den Jahren nach der Behandlung

Insbesondere auch bei einer geplanten Zulassung zur Behandlung von Kindern („Kinderindikation“) ist die Durchführung von klinischen Studien zum Nachweis der Wirksamkeit und Sicherheit in der entsprechenden Patientengruppe notwendig. Bei Kindern und Jugendlichen handelt es sich um eine Personengruppe mit eigenen Erfordernissen, die für die Arzneimittelentwicklung gesetzlich verankert sind (Verordnung (EG) Nr. 1901/2006 vom 12.12.2006) [22, 23]. Seit ihrem Inkrafttreten im Januar 2007 [22] muss ein pharmazeutisches Unternehmen für jedes neue Arzneimittel auch ein pädiatrisches klinisches Prüfprogramm auflegen oder begründen, warum dies nicht möglich oder nicht erforderlich ist.

Dies gilt auch für AIT-Produkte. Die Entwicklung von sicheren und wirksamen AIT-Produkten zum Einsatz bei Kindern und Jugendlichen erfordert die Durchführung von entsprechenden klinischen Studien. Diese erfolgen zeitlich versetzt zu den klinischen Untersuchungen bei Erwachsenen, wenn bereits aus diesen Erfahrungen zu Wirksamkeit und Sicherheit vorliegen. Es wird dabei eine Datengrundlage angestrebt, die mittelfristig eine Extrapolation von Studienergebnissen von Erwachsenen auf Kinder und Jugendliche zulässt [24]. Dafür ist es notwendig, insbesondere zu krankheitsmodifizierenden Langzeiteffekten der AIT-Behandlung eine Datenbasis zu schaffen. So hat jedes pharmazeutische Unternehmen die Langzeitwirksamkeit bei Erwachsenen und zeitlich um ein Jahr versetzt bei Kindern für *ein* von ihm gewähltes AIT-Produkt aus dem Gesamtportfolio der Firma zu untersuchen. Details finden sich in der derzeit gültigen Version des durch das Pediatric Committee (PDCO) der Europäischen Arzneimittelagentur (EMA) genehmigten pädiatrischen Prüfplans (Standard PIP; [25]). Wird für dieses Referenzprodukt eine Langzeitwirksamkeit gezeigt, müssen für die anderen Produkte des Herstellers keine weiteren Langzeitstudien nach PIP durchgeführt werden. Diese wenigen Langzeitstudien, die parallel bei Erwachsenen und zeitlich versetzt bei Kindern und Jugendlichen mit demselben Produkt durchgeführt werden, sind notwendig, um die bestehende wissenschaftliche Lücke zu schließen und den Nachweis eines ähnlichen, krankheitsmodifizierenden Therapieeffektes bei Kindern und Erwachsenen zu erbringen, der dann zukünftige Extrapolationen ermöglicht [24].

### Wissenschaftliche Leitlinien und indikationsgemäßer AIT-Einsatz

Der Einsatz der in Deutschland zugelassenen AIT-Produkte dient der kausalen Behandlung von IgE-vermittelten Allergien und Prävention von Krankheitsprogression [11, 15]. Es handelt sich um zugelassene First-Line-Therapien, die ihre überlegene klinische Wirksamkeit im Zulassungsverfahren gegenüber Placebo in einem randomisierten, placebokontrollierten Doppelblinddesign belegen müssen [21, 26]. Sie enthalten keine Restriktionen ihres Einsatzes im Sinne von Second- oder Third-Line-Therapeutika z. B. nur bei Versagen einer pharmakologischen symptomatischen Therapie [27]. Die Besonderheit des direkten Facharztzugangs im deutschen Gesundheitssystem ermöglicht eine frühzeitige indikationsgerechte Nutzung der AIT unter Ausnutzung krankheitsmodifizierender Effekte dieser Therapieform [11, 27].

### Wissenschaftliche Leitlinien und produktspezifische Kontraindikationen

Die produktspezifische Bewertung der im Zulassungsdossier vorgelegten Daten erfolgt im Rahmen des Zulassungsverfahrens durch die zuständige Behörde, basierend auf dem jeweils aktuellen Stand der Wissenschaft, geltenden Gesetzen und regulatorischen Leitlinien. Neue wissenschaftliche Erkenntnisse finden dabei entsprechende Berücksichtigung. Beispielsweise erfolgte in der aktuellen europäischen Leitlinie zur Behandlung von Insektengiftallergien [17], der eine evidenzbasierte systematische Datenauswertung zugrunde liegt, eine Neubewertung der seit Langem für die Insektengiftimmuntherapie bestehenden Kontraindikation (KI) der Einnahme von Betablockern oder ACE-Hemmern: Die neuen Leitlinienempfehlungen sprechen sich dafür aus, dass diese Medikamente nach entsprechender Aufklärung über ein potenziell erhöhtes Risiko für starke Nebenwirkungen unter AIT mit Insektengiften weiter eingenommen werden können. Dies führte (vorübergehend) zu einer Diskrepanz zwischen den Fachinformationen der in Deutschland zugelassenen Insektengifttherapieallergene und der europäischen Leitlinie bezüglich Betablockern und ACE-Hemmern als KI, was in Fachkreisen die medizinrechtliche Frage aufwarf, ob Leitlinie oder Fachinformation bindend ist [28]. Entscheidend ist der Inhalt der Fachinformation. Leitlinien haben in diesem Sinne allenfalls beratende Bedeutung und können nicht die Aussagen der Fachinformation relativieren oder aufheben. Die Anwendung eines Insektengiftproduktes, das die entsprechenden KI in den Gegenanzeigen enthält, stellt bei Patienten unter Betablockertherapie einen „off label use“ dar [28].

Basierend auf den neuen wissenschaftlichen Erkenntnissen, die der o. g. Leitlinie [17] zugrunde liegen, ist es jedoch möglich, eine zustimmungspflichtige Änderungsanzeige (in der Regel Typ-II-Variation) zur bestehenden Zulassung bei der zuständigen Zulassungsbehörde zu stellen, die nach entsprechender wissenschaftlicher Bewertung und Genehmigung ermöglicht, die Angaben zu besagten KI in der jeweiligen Fachinformation zu ändern.

Zwischenzeitlich wurden entsprechende Änderungsanzeigen von 2 der 3 Hersteller der in Deutschland zugelassenen Insektengiftpräparate gestellt und bewilligt: Die Ausführungen zu Betablockern und ACE-Hemmern sind bei den betreffenden Produkten jetzt unter 4.4 (Besondere Warnhinweise und Vorsichtsmaßnahmen für die Anwendung) bzw. 4.5 (Wechselwirkungen mit anderen Arzneimitteln und sonstige Wechselwirkungen) der Fachinformation aufgeführt.

## Regulatorische Voraussetzungen

### Gesetzliche Rahmenbedingungen für die Zulassung von AIT-Produkten

Allergene unterliegen seit 1989 europäischem Recht (Richtlinie 89/342/EWG; [29]). Nach der Definition der Richtlinie 2001/83/EG [23] sind sowohl Test- als auch Therapieallergene Arzneimittel. Nach Artikel 6 dieser europäischen Richtlinie darf ein Arzneimittel in einem Mitgliedstaat erst dann in den Verkehr gebracht werden, wenn die zuständige Behörde dieses Mitgliedstaats eine Genehmigung dafür erteilt hat. Alle EU-Mitgliedstaaten verfügen über mindestens eine eigene nationale Zulassungsbehörde, die im Netzwerk der europäischen Arzneimittelbehörden oder unter EMA-Koordination kooperieren [30].

In Deutschland ist der Anwendungsbereich der Richtlinie 2001/83/EG im Arzneimittelgesetz (AMG; [31]) umgesetzt. Gemäß § 21 Abs. 1 AMG dürfen Arzneimittel in Deutschland nur in Verkehr gebracht werden, wenn sie von der zuständigen Bundesoberbehörde zugelassen worden sind. Für die Zulassung muss nach dem jeweils aktuellen Stand des Wissens belegt werden, dass die Arzneimittel eine angemessene *Qualität* besitzen sowie *wirksam* und *sicher* sind. Zuständige Bundesoberbehörde für Test- und Therapieallergene ist das Paul-Ehrlich-Institut (PEI) mit Sitz in Langen bei Frankfurt/Main (www.pei.de), in dessen Zuständigkeitsbereich Impfstoffe und biomedizinische Arzneimittel fallen [30].

Die Anforderungen an die Zulassung eines Arzneimittels (somit auch für Test- und Therapieallergene) sind innerhalb der EU harmonisiert und gelten daher sowohl für die Bundesrepublik Deutschland als auch für alle weiteren Mitgliedstaaten (MS) der EU [32]. In der EU bestehen prinzipiell 4 verschiedene Verfahren, um ein Arzneimittel zuzulassen [30, 32]:

- nationales Zulassungsverfahren: Zulassung in einem MS,
- Mutual Recognition Procedure (MRP, Verfahren der gegenseitigen Anerkennung): Ausweitung einer in einem MS bestehenden Zulassung auf einen weiteren oder mehrere MS,
- Decentralized Procedure (DCP, dezentrales Zulassungsverfahren): gleichzeitige Zulassung in mehreren MS,
- Centralized Procedure (CP, zentrales Zulassungsverfahren): gleichzeitige Zulassung in allen MS.

Nicht nur bei nationalen, sondern auch bei multinationalen Zulassungsverfahren kommt den nationalen Arzneimittelbehörden als Reference Member State, Concerned Member State, Rapporteur oder Co-Rapporteur eine wesentliche Aufgabe bei der Bewertung von Zulassungsdossiers im Hinblick auf Qualität, Sicherheit und Wirksamkeit zu [30].

### Anforderungen im Zulassungsverfahren

Grundsätzlich müssen für jedes Arzneimittel eine ausreichende Qualität, Sicherheit und Wirksamkeit im Zulassungsverfahren belegt werden und das sogenannte Nutzen-Risiko-Verhältnis muss positiv sein [23]. Hierbei entspricht im Wesentlichen die Wirksamkeit des jeweiligen Arzneimittels dem anzurechnenden Nutzen; die Nebenwirkungen oder eine mangelnde/nicht vorhandene Wirksamkeit entsprechen dem gegebenen Risiko. Sollte die Wirksamkeit eines Produktes nicht oder nicht ausreichend belegt werden, kann bereits aus diesem Grund kein positives Nutzen-Risiko-Verhältnis vorliegen.

Die spezifischen Anforderungen ergeben sich aus den gesetzlichen Vorgaben (unter anderem aus dem deutschen AMG [31], bspw. § 22 ff.), dem Europäischen Arzneibuch [33] sowie anerkannten wissenschaftlichen Leitlinien (bspw. EMA Guideline on Allergen Products: Production and Quality Issues (CHMP/BWP/304831/07) [34]). Dabei ist zu beachten, dass es sich bei Therapieallergenen um biologische Arzneimittel handelt. Die Eigenschaften derartiger Arzneimittel bezüglich ihrer Qualität, Sicherheit und Wirksamkeit sind in hohem Maße davon abhängig, wie diese Arzneimittel vom jeweiligen pharmazeutischen Unternehmen hergestellt und kontrolliert werden. Insbesondere die Herstellung ist jedoch für jedes Produkt verschiedener Hersteller unterschiedlich, sodass hier keine Übertragbarkeit von Daten zur Qualität, Sicherheit oder Wirksamkeit zwischen Arzneimitteln vorgenommen werden kann. Diese Einschätzung wurde durch zahlreiche klinische Studien bestätigt, die klar belegen, dass diese Faktoren nur individuell *produktspezifisch* bewertet werden können [10]: Wenn ein AIT-Produkt nachweislich wirksam ist, kann sich dies bei einem anderen AIT-Produkt, das gegen dieselbe Allergenquelle gerichtet ist, aber einem anderen Herstellungsgang unterzogen wurde, völlig anders verhalten. Nur in sehr begrenzten Ausnahmefällen, beispielsweise wenn Arzneimittel verwandte (kreuzreaktive) Allergene enthalten, die Teil einer sogenannten homologen Gruppe sind (siehe EMA-Leitlinie *Allergen products: production and quality issues* (CHMP/BWP/304831/07) [34]), und bestimmte weitere Anforderungen erfüllt sind (u. a. gleiche Herstellung, gleiche Darreichungsform), kann eine Übertragung bestimmter klinischer Daten erfolgen. Die allgemeinen Anforderungen an klinische Studien für Therapieallergene sind in der EMA *Guideline on the clinical development of products for specific immunotherapy for the treatment of allergic diseases* (CHMP/EWP/18504/2) dargelegt [21].

Des Weiteren befindet sich eine Leitlinie in Ausarbeitung, die speziell auf die Entwicklung von Allergenprodukten ausgerichtet ist, die nur kleine Patientenpopulationen betreffen (*Concept paper on a guideline for allergen products development in moderate to low-sized study-populations;* [35]). Diese Leitlinie soll sowohl Therapie- als auch Testallergene behandeln.

### Ausnahme von der Zulassungspflicht (Individualrezepturen)

Nach der europäischen Richtlinie 2001/83/EG sind Ausnahmen von der Zulassungspflicht für Arzneimittel nach *Artikel 5 *(Richtlinie 2001/83/EG; [23]) möglich: „Ein Mitgliedstaat kann gemäß den geltenden Rechtsbestimmungen in besonderen Bedarfsfällen Arzneimittel von den Bestimmungen der Richtlinie ausnehmen, … die nach den Angaben eines zugelassenen praktizierenden Arztes hergestellt werden und zur Verabreichung an dessen eigene Patienten unter seiner unmittelbaren persönlichen Verantwortung bestimmt sind“ (Individualrezepturen). Auch das in Deutschland gültige AMG enthält nach § 21 (2) eine Ausnahmeregelung [31]: „Einer Zulassung bedarf es nicht für Arzneimittel, die … § 21 (2), 1g: als Therapieallergene für einzelne Patienten aufgrund einer Rezeptur hergestellt werden“. Diese Ausnahmeregelung ist sinnvoll und wichtig für die Verfügbarkeit von allergenspezifischen Immuntherapien für Allergien auf *seltene* Allergenquellen [30]. Nachdem in den MS die Auslegung und Umsetzung von Artikel 5 (Richtlinie 2001/83/EG) sehr uneinheitlich erfolgte, erscheinen gegenwärtig die regulatorischen Rahmenbedingungen für die Zulassung und das Inverkehrbringen von Allergenpräparaten in der EU inhomogen. Die Durchdringung der Marktsituation bezüglich der Verfügbarkeit von bestimmten Allergenpräparaten und eine gegenseitige Anerkennung (MRP-Verfahren) in verschiedenen EU-Ländern ist dadurch erschwert. Die Europäische Kommission hat daher die *Coordination Group for Mutual Recognition and Decentralised Procedures – Human (CMDh)* damit beauftragt, eine Harmonisierung auf europäischer Ebene anzustreben. Eine entsprechende Guideline (*Recommendations on common regulatory approaches for allergen products* CMDh/399/2019, Rev. 0 (July 2020)) wurde im CMDh unter Federführung des PEI erarbeitet und nach erfolgreichem Freigabeverfahren kürzlich publiziert [36].

### Therapieallergene-Verordnung (TAV)

Die TAV, seit 14.11.2008 in Deutschland in Kraft, stellt sicher, dass die o. g. Ausnahmeregelung nach AMG § 21 (2), 1g nicht auf AIT-Produkte für die in Deutschland *häufigen* Allergenquellen (Süßgräser ohne Mais sowie auf Birke, Erle, Hasel, Hausstaubmilben, Bienengift, Wespengift) angewandt werden kann.

Für diese *häufigen* Therapieallergene müssen in Deutschland die Qualität, Wirksamkeit und Sicherheit belegt und in einem Zulassungsverfahren überprüft werden.

Für die zum Zeitpunkt des Inkrafttretens der TAV auf dem Markt befindlichen Individualrezepturen (*n* = 6654), die eine oder mehrere der aufgeführten Allergenquellen enthielten, mussten die pharmazeutischen Unternehmen entscheiden, ob sie eine Zulassung anstreben oder nicht [37]. Bis zum Stichtag (01.12.2010) wurden Zulassungsanträge für 123 dieser Individualrezepturen, die unter die TAV fallen, im PEI eingereicht. Im Rahmen der gesetzlichen Übergangsfristen der TAV muss für diese verkehrsfähigen Produkte der Nachweis von Qualität, Sicherheit und Wirksamkeit gemäß der o. g. Anforderungen im Zulassungsverfahren erbracht werden [38]. Zusätzlich unterliegen besagte „TAV-Produkte“ der staatlichen Chargenprüfung. Dies unterscheidet sie von regulären Individualrezepturen mit Wirkstoffen anderer (als der o. g., nach TAV geregelten) Allergenquellen. Letztere unterliegen nicht der staatlichen Chargenprüfung, sondern generellen Anforderungen der Good Manufacturing Practice (GMP), die von pharmazeutischen Unternehmen eingehalten werden müssen.

Sollten aufgrund der im laufenden TAV-Verfahren erhobenen klinischen Studiendaten begründete Zweifel an der Wirksamkeit eines Produktes bestehen, werden keine weiteren Chargen durch das PEI freigegeben. Sollten in einer klinischen Prüfung die primären Endpunkte nicht erreicht worden sein, sind neben einer möglichen mangelnden Wirksamkeit weitere Faktoren zu prüfen (bspw. Schwächen im Studiendesign, Rekrutierungsproblematik geeigneter Probanden, Probleme in der Studiendurchführung, Pollenflug), die hierfür ausschlaggebend sein können. Eine entsprechende Bewertung kann ausschließlich nach Prüfung der jeweiligen Unterlagen zu den betreffenden klinischen Studien erfolgen. Das PEI ist dazu verpflichtet, die Unterlagen und Daten von Antragstellern vertraulich zu behandeln, und kann somit grundsätzlich keine Informationen zu noch im Verfahren befindlichen Zulassungsanträgen an Dritte geben. Vorgaben zur Information der Öffentlichkeit ergeben sich insbesondere aus § 34 (AMG). Gemäß diesen Vorgaben ist auch keine rechtliche Grundlage gegeben, wonach das Versagen von Chargenfreigaben (im Gegensatz zu einer Rücknahme oder eines Widerrufs einer Chargenfreigabe) öffentlich kommuniziert werden dürfte, weshalb von außen die vom PEI ergriffenen Maßnahmen mitunter nicht unmittelbar wahrgenommen werden können.

Produkte, für die vom Hersteller zum Stichtag keine Zulassung angestrebt wurde, waren noch bis 14.11.2011 verkehrsfähig, danach nicht mehr, da zu diesem Zeitpunkt die Übergangsfrist für das Inverkehrbringen endete [37, 38].

## AIT-Produkte im deutschen Markt

Aktuell (Stand: 01.07.2020) sind in Deutschland 62^1^ AIT-Produkte zugelassen zur Behandlung von Typ-I-Allergien (Tab. 1A), davon 55 ab einem Lebensalter von 5 Jahren, ein Produkt ab 12 Jahren, 6 Produkte ab 18 Jahren [39]. Dies umfasst Therapieallergene gegen Birke, Erle, Hasel, Frühblühermischungen, Gräsermischungen, Gräsermischung/Birke, Gräser/Bäumemischung, Gräsermischung/Roggen, Gräsermischung/Getreide, verschiedene Gräsermischungen/Roggen oder Getreide/Birke oder Beifuß u./o. Wegerich, Ragweed (Traubenkraut), Beifuß, Wegerich, Glaskraut, *Dermatophagoides* (*D.*) *pteronyssinus, D. farinae*, Milbenmischungen, Bienengift, Wespengift.

Die Mehrheit (*n* = 53) der zugelassenen 62 AIT-Produkte erhielt die Zulassung vor 2005 in einem nationalen Zulassungsverfahren. Nach 2005 erhielten in Deutschland 4 AIT-Produkte ihre Zulassung in einem nationalen Zulassungsverfahren, 3 Produkte in einem dezentralen europäischen Zulassungsverfahren (DCP) und 2 Produkte im Rahmen der gegenseitigen Anerkennung (MRP).

Neben den o. g. zugelassenen AIT-Produkten befinden sich 61 Therapieallergene (gegen Birke, Frühblühermischung (Birke/Erle/Hasel), Wiesenlieschgras, Gräsermischung, Wiesenlieschgras/Roggen, Gräsermischung/Roggen, Gräsermischung/Birke, Gräsermischung/Roggen und Beifuß, Gräsermischung/Roggen und Birke/Erle/Hasel, Gräsermischung/Bäumemischung, Milbenmischung, D. farinae, D. pteronyssinus) derzeit gemäß der gesetzlichen Übergangsfristen in einem laufenden Zulassungsverfahren unter der TAV (Tab. 1B) und sind bis zur Entscheidung über die Zulassung verkehrsfähig [38, 40].

Der aktuelle Stand der in Deutschland zugelassenen Therapieallergene und verkehrsfähigen Therapieallergene unter der Übergangsvorschrift der TAV ist jeweils der PEI-Homepage zu entnehmen. Die Liste erlaubt jedoch keine Rückschlüsse über die tatsächliche Verfügbarkeit der Produkte oder, ob für diese Produkte seitens des PEI Chargenfreigaben erteilt werden.

## Neue Entwicklungen

### Neue Wirkstoffe

#### Molekulare Allergenkomponenten und Peptide in der AIT

Bezüglich Potenzial und Limitationen von hochreinen nativen Allergenen, rekombinanten Allergenen und synthetischen Peptiden und ihren Einsatz in klinischen Phase-II- und Phase-III-Studien wird auf den Beitrag von Holzhauser et al. in diesem Themenheft verwiesen. Bislang hat kein gereinigtes Allergenmolekül oder ein molekular definiertes Allergenderivat ein Zulassungsverfahren in Deutschland oder einem anderen EU-Mitgliedstaat durchlaufen. Regulatorisch sind unter gereinigten Allergenen alle reinen Allergene zu verstehen, unabhängig davon, ob sie aus nativen Extrakten isoliert oder durch rekombinante DNA-Technologie hergestellt wurden [21]. Darüber hinaus werden regulatorisch auch chemisch synthetisierte Peptide, die von Allergensequenzen abgeleitet sind, als gereinigte Allergenkomponenten betrachtet [21]. In bisherigen Phase-III-Studien waren die Ergebnisse im Hinblick auf eine klinische Wirksamkeit unterschiedlich [39].

Aber selbst erfolgreiche Studien, die eine klinische Wirksamkeit der rekombinanten AIT-Produkte im Symptom-Medikations-Score (SMS) während der Pollenflugzeit zeigten, erzielten in vergleichenden Kopf-an-Kopf-Studien keinen größeren Behandlungseffekt im Vergleich zu konventionellen Extrakten, jedoch waren verkürzte Behandlungsschemata möglich [41]. In den bislang durchgeführten klinischen Studien mit rekombinanten Allergenen erfolgte durch die Auswahl einzelner oder mehrerer Allergenkomponenten, die anhand ihrer auf Publikationen und zusätzlichen Untersuchungen der jeweiligen Hersteller abgeleiteten Bedeutung ausgewählt wurden, die Behandlung gegenüber natürlichen Allergenextrakten mit einem eingeschränkten Allergenspektrum. Möglicherweise ist das Potenzial einer Allergenkomponenten-basierten AIT in den bisherigen Ansätzen noch nicht vollständig ausgeschöpft. Patienten weisen ein individuelles Sensibilisierungsmuster gegenüber Allergenen einer Allergenquelle auf, die für die allergische Erkrankung bei einzelnen Personen relevant sind [21].

Während das Konzept einer komponentenbasierten Allergiediagnostik basierend auf gereinigten Allergenkomponenten (z. B. molekulare Allergiediagnostik, „component-resolved diagnostics“, CRD) Eingang in die Praxis der allergologischen Routinediagnostik gefunden hat [42, 43], konnte das Konzept einer komponentenbasierten AIT („component-resolved immunotherapy“, CRIT; [44]), bei der die Allergene individuell nach dem Sensibilisierungsprofil des Patienten zur Behandlung zusammengestellt werden, nicht verwirklicht werden. Dies mag nicht zuletzt an regulatorischen Erfordernissen gelegen haben. In den letzten Jahren hat sich jedoch die regulatorische Sicht in Bezug auf personalisierte Therapieansätze insbesondere durch Entwicklungen in der Onkologie stark verändert: In diesem Bereich werden bereits einzelne Moleküle, die nach Good Manufacturing Practice (GMP) hergestellt werden, individuell für einzelne Patienten gemischt (personalisierte Medizin) und im Rahmen von Korb- und Dachstudien angewandt [41]. Ähnliche Ansätze im Bereich der Allergie sind nun aus regulatorischer Sicht hypothetisch möglich [41].

#### Nahrungsmittelallergene in der AIT

In zahlreichen wissenschaftlichen Untersuchungen wurden Ansätze der AIT zur Behandlung von IgE-vermittelten Nahrungsmittelallergien verfolgt. Es wurden überwiegend orale Immuntherapie (OIT), aber auch sublinguale (SLIT) und epikutane (EPIT) Verabreichungsformen eingesetzt. Die Ergebnisse einer systematischen Datenauswertung wurden in einer aktuellen Leitlinie zusammengefasst [19]. Wissenschaftliche Schlüsselerkenntnisse [19, 45] sind:

- AIT bei Lebensmittelallergien sollte aufgrund von potenziellen allergischen Nebenwirkungen nur von erfahrenem Personal in klinischen Zentren mit umfangreicher Vorerfahrung in diesem Bereich durchgeführt werden.
- AIT gegen Nahrungsmittelallergie sollte in Erwägung gezogen werden bei Kindern im Alter ab ca. 4–5 Jahren mit einer persistierenden IgE-vermittelten Nahrungsmittelallergie gegen Kuhmilch, Hühnerei oder Erdnuss zur Erhöhung der Reaktionsschwelle unter Therapie.
- Ein anhaltender Effekt nach Beendigung der Therapie wird angenommen, ist aber nicht belegt.
- Die orale Immuntherapie (OIT) bietet eine bessere Wirksamkeit als die SLIT, jedoch ist die OIT mit einer höheren Häufigkeit von unerwünschten Ereignissen assoziiert als SLIT, obwohl die meisten nicht schwerwiegend sind.
- Die Anfangsdosis und jede Dosissteigerung während der Aufbauphase sollte im klinischen Umfeld verabreicht werden.
- Patienten und ihre Familien sollten über die Anwendung der AIT bei IgE-vermittelten Nahrungsmittelallergien informiert werden, damit sie eine wohlüberlegte Therapieentscheidung treffen können.
- Prospektive, gut konzipierte Längsschnittstudien sind erforderlich, um die zahlreichen Erkenntnislücken zu schließen mit dem Ziel, AIT-Protokolle in der klinischen Praxis als Routinetherapie für Nahrungsmittelallergien zu implementieren.
- Es gibt nur wenige Belege für den erfolgreichen Einsatz von AIT gegen Nahrungsmittelallergie bei Erwachsenen.

In klinischen Studien mit Erdnussallergenen (OIT und EPIT) sprachen Erwachsene und Jugendliche in der Regel schlechter an als Kinder [46, 47], was Hinweis auf ein unterschiedliches Ansprechen des Immunsystems von Erwachsenen und Kindern gibt. Dies verdeutlicht, dass eine Extrapolation von Kindern auf Erwachsene und *vice versa* nicht ohne entsprechende Datenlage aus parallel durchgeführten klinischen Studien möglich ist (s. oben „Kinderindikation“; [24, 25]).

Eines der beiden o. g. Erdnussprodukte (AR101; [46]), das als aktiven Wirkstoff einen standardisierten Erdnusspuder enthält, wurde kürzlich basierend auf den Ergebnissen eines Entwicklungsprogramms mit 2 randomisierten doppelblinden, placebokontrollierten klinischen Phase-III-Studien in den USA zur OIT der Erdnussallergie bei Kindern und Jugendlichen mit Erdnussallergie von der Food and Drug Administration (FDA) zugelassen [8]. Dasselbe Produkt befindet sich derzeit in einem zentralen europäischen Zulassungsverfahren mit dem PEI als Rapporteur. Im Falle einer positiven Nutzen-Risiko-Bewertung und eines entsprechenden positiven Abschlusses des Zulassungsverfahrens würde es sich um das erste Therapieallergen handeln, das in einem zentralen Zulassungsverfahren gleichzeitig eine Zulassung in allen europäischen MS erhält. Weitere Nahrungsmittelallergene wurden in klinischen Entwicklungsprogrammen in Phase-II- und -III-Studien mit unterschiedlichem Erfolg eingesetzt [19]. Es handelt sich bei Nahrungsmittelallergenen regulatorisch um eine neue Wirkstoff- und Produktklasse, für die noch keine spezifische EMA-Leitlinie existiert. Die bereits erwähnte EMA-Leitlinie zur klinischen Entwicklung von AIT-Produkten [21] adressiert bereits die Bedeutung von IgE-vermittelten Nahrungsmittelallergien als wichtige Ursache für akute Anaphylaxie und anaphylaxiebedingte Todesfälle und weist auf den bestehenden Mangel einer etablierten kausalen Behandlung hin [21]. Dem Potenzial laufender klinischer Studien zur AIT von Nahrungsmittelallergien wurde Rechnung getragen durch Aufnahme der spezifischen Immuntherapie für Nahrungsmittelallergie in das 7. Rahmenprogramm für Forschung und Entwicklung der Europäischen Union [21]. Die bestehende EMA-Leitlinie umreißt orientierend eine Herangehensweise für zukünftige Zulassungsverfahren [21]: Zur Diagnosestellung und für die Bewertung der Wirksamkeit der Behandlung wird als Goldstandard die Durchführung einer doppelblinden, placebokontrollierten Nahrungsmittelprovokation (DBPCFC) zur Identifikation der symptomlos vertragenen Nahrungsmitteldosis definiert [21]. Aufgrund der Tatsache, dass die spezifische Verabreichung von Allergenen an Patienten mit bestehender Nahrungsmittelallergie ein hohes Risiko für allergische Reaktionen birgt und nur begrenzte Erfahrungen mit der AIT bei Nahrungsmittelallergien vorliegen, wird empfohlen, für entsprechende klinische Studien fallbezogen individuelle wissenschaftliche Beratung bei den zuständigen Behörden einzuholen [21]. Dieser Vorgehensweise wurde bei beiden genannten Erdnussprodukten gefolgt. Die regulatorische Bewertung erfolgt hierbei auf dem Stand der wissenschaftlichen Erkenntnisse. Als primäre Endpunkte wurden die nach Behandlung im DBPCFC vertragenen Einzeldosen im Vergleich zum Ausgangsbefund vor Behandlung definiert, wobei ein Responder im europäischen Zulassungsverfahren durch eine vertragene Einzeldosis von 1000 mg (EU), im amerikanischen Verfahren von 600 mg (USA) definiert wird. Bezüglich der Therapiedauer ist bisher kein Zeitraum definiert. Im Gegensatz zur AIT für allergische Rhinitis fehlt derzeit die Evidenz, dass AR101 nach Absetzen der OIT eine Langzeittoleranz induzieren kann [8].

### Neue Adjuvanzien

Neben dem aktiven Wirkstoff sind Adjuvanzien für die Wirkung von AIT-Produkten von Bedeutung. Während zahlreiche Adjuvanzien als interessante Kandidaten für die AIT in klinischen Prüfungen untersucht werden [48, 49] und weitere aus der Impfstoffentwicklung ein Potenzial für einen Einsatz in der AIT haben könnten [50], werden derzeit 3 Adjuvanzien in den in Deutschland zugelassenen/verkehrsfähigen AIT-Produkten eingesetzt: Aluminiumhydroxid Al(OH)_3_ ist das am häufigsten verwendete Adjuvans, während mikrokristallines Tyrosin (MCT) und Monophosphoryl-Lipid‑A (MPL) weniger häufig verwendet werden (Tab. 1; [48]). Gegenwärtig befinden sich keine AIT-Produkte mit anderen als den 3 genannten Adjuvanzien in einem Zulassungsverfahren für den deutschen Markt. Das Potenzial jedes einzelnen Adjuvans für die AIT muss individuell bewertet werden. Dabei kann insbesondere ihre Sicherheitsbewertung aufgrund der extrem unterschiedlichen Art der Substanzen (bezüglich Herkunft, Zusammensetzung, Herstellung, Antigenität) und unterschiedlicher Funktions‑/Aktionsweisen eine besondere regulatorische Herausforderung darstellen. Aufgrund dessen sind teilweise verschiedene Guidelines heranzuziehen (z. B. [51, 52]). Bei der Bewertung ist insbesondere zu berücksichtigen, dass im Gegensatz zu Impfstoffen die Applikation von adjuvantierten AIT-Produkten über mehrere Jahre hinweg repetitiv erfolgt.

## Fazit

Erkrankungen durch Soforttypallergien zeigen in Deutschland bei Erwachsenen, Jugendlichen und Kindern eine hohe Prävalenz mit zunehmender Tendenz bezüglich Asthma bronchiale. Sie sind mithilfe einer AIT kausal behandelbar. Nach aktuellen Zahlen des Robert Koch-Instituts erhalten derzeit nur 30 % der Kinder und Jugendlichen in der Altersgruppe der 11- bis 17-Jährigen mit ärztlicher Heuschnupfen- oder Neurodermitisdiagnose nach einem positiven Allergietest eine AIT. Im Gegensatz zu zahlreichen anderen europäischen Ländern ist in Deutschland für Betroffene ein unmittelbarer Zugang zu allergologisch versierten Facharztgruppen und die frühzeitige Einleitung einer AIT möglich. Zugelassene und verkehrsfähige AIT-Produkte zur subkutanen oder sublingualen AIT stehen zur Behandlung von Allergien gegen häufige Allergenquellen (Birke, Erle, Hasel, Gräser, Hausstaubmilben, Biene und Wespengift) und verschiedene Kräuter als Fertigprodukte zur Verfügung. Daneben besteht für seltene Allergenquellen die Möglichkeit der Individualrezeptur. Das Spektrum der mittels AIT kausal behandelbaren Erkrankungen könnte bei positivem Nutzen-Risiko-Verhältnis in absehbarer Zeit auch Nahrungsmittelallergien betreffen; ein erstes OIT-Produkt zur Behandlung der Erdnussallergie wird aktuell in einem zentralen europäischen Zulassungsverfahren auf Qualität, Wirksamkeit und Sicherheit überprüft. Bei der Entwicklung von AIT-Produkten – insbesondere in einem neuen Indikationsgebiet, wie z. B. Asthmaprävention und -behandlung oder mit Nahrungsmittelallergenen als neuer Wirkstoffklasse – sind klinische Forschung und behördliche Regulation eng verzahnt und erfahren idealerweise eine gemeinsame Weiterentwicklung.

## Footnotes

1 Mit Doubletten und unterschiedlichen Stärken/Packungsgrößen: 71.

## References

[1] Klimek L, Vogelberg C, Werfel T, Klimek L, Vogelberg C, Werfel T (2019). Weißbuch Allergie in Deutschland.

[2] Thamm R, Hey I, Thamm M, Klimek L, Vogelberg C, Werfel T (2019). Epidemiologie allergischer Erkrankungen: Prävalenzen und Trends in Deutschland. Weißbuch Allergie in Deutschland.

[3] Robert Koch-Institut (2015). Gesundheit in Deutschland. Gesundheitsberichterstattung des Bundes. Gemeinsam getragen von RKI und Destatis.

[4] Thamm R, Poethko-Müller C, Hüther A, Thamm M (2018). Allergische Erkrankungen bei Kindern und Jugendlichen in Deutschland – Querschnittergebnisse aus KiGGS Welle 2 und Trends. J Health Monit.

[5] Poethko-Müller C, Thamm M, Thamm R (2018). Heuschnupfen und Asthma bei Kindern und Jugendlichen in Deutschland – Querschnittergebnisse aus KiGGS Welle 2 und Trends. J Health Monit.

[6] Mahler V, Esch RE, Kleine-Tebbe J (2019). Understanding differences in allergen immunotherapy products and practices in North America and Europe. J Allergy Clin Immunol.

[7] Shamji MH, Durham SR (2017). Mechanisms of allergen immunotherapy for inhaled allergens and predictive biomarkers. J Allergy Clin Immunol.

[8] Lam HY, Tergaonkar V, Ahn KS (2020). Mechanisms of allergen-specific immunotherapy for allergic rhinitis and food allergies. Biosci. Rep..

[9] Arzt L, Bokanovic D, Schrautzer C (2018). Immunological differences between insect venom-allergic patients with and without immunotherapy and asymptomatically sensitized subjects. Allergy.

[10] Vercelli D (2010). Gene-environment interactions in asthma and allergy: the end of the beginning?. Curr Opin Allergy Clin Immunol.

[11] Pfaar O, Bachert C, Bufe A (2014). Guideline on allergen-specific immunotherapy in IgE-mediated allergic diseases: S2k guideline of the German society for allergology and clinical immunology (DGAKI), the society for pediatric allergy and environmental medicine (GPA), the medical association of German Allergologists (AeDA), the Austrian society for allergy and immunology (ÖGAI), the Swiss society for allergy and immunology (SGAI), the German society of dermatology (DDG), the German society of Oto-rhino-laryngology, head and neck surgery (DGHNO-KHC), the German society of pediatrics and adolescent medicine (DGKJ), the society for pediatric Pneumology (GPP), the German respiratory society (DGP), the German association of ENT surgeons (BV-HNO), the professional federation of Paediatricians and youth doctors (BVKJ), the federal association of Pulmonologists (BDP) and the German dermatologists association (BVDD). Allergo J Int.

[12] Shamji MH, Kappen JH, Akdis M (2017). Biomarkers for monitoring clinical efficacy of allergen immunotherapy for allergic rhinoconjunctivitis and allergic asthma: an EAACI Position Paper. Allergy.

[13] Sindher SB, Long A, Acharya S, Sampath V, Nadeau KC, Parker SN (2018). The use of biomarkers to predict aero-allergen and food immunotherapy responses HHS Public Access. Clin Rev Allergy Immunol.

[14] Pfaar O, Gerstlauer M, Saloga J, Vogelberg C, Kleine-Tebbe J, Klimek L, Vogelberg C, Werfel (2019). Allergen-Immuntherapie (Hyposensibilisierung). Weißbuch Allergie in Deutschland.

[15] Roberts G, Pfaar O, Akdis CA (2018). EAACI guidelines on allergen immunotherapy: allergic rhinoconjunctivitis. Allergy.

[16] Agache I, Lau S, Akdis CA (2019). EAACI guidelines on allergen immunotherapy: house dust mite-driven allergic. Asthma.

[17] Sturm GJ, Varga EM, Roberts G (2018). EAACI guidelines on allergen immunotherapy: hymenoptera venom allergy. Allergy.

[18] Halken S, Larenas-Linnemann D, Roberts G (2017). EAACI guidelines on allergen immunotherapy: prevention of allergy. Pediatr Allergy Immunol.

[19] Pajno GB, Fernandez-Rivas M, Arasi S (2018). EAACI Guidelines on allergen immunotherapy: IgE-mediated food allergy. Allergy.

[20] EMA (2001) Points to consider on application with 1. Meta-analyses; 2. One pivotal study (CPMP/EWP/2330/99). https://www.ema.europa.eu/en/documents/scientific-guideline/points-consider-application-1meta-analyses-2one-pivotal-study_en.pdf. Zugegriffen: 6. Juni 2020

[21] EMA (2008). Guideline on the clinical development of products for specific immunotherapy for the treatment of allergic diseases (CHMP/EWP/18504/2006).

[22] Verordnung (EG) Nr. 1901/2006 des Europäischen Parlaments und des Rates vom 12. Dezember 2006 über Kinderarzneimittel und zur Änderung der Verordnung (EWG) Nr. 1768/92, der Richtlinien 2001/20/EG und 2001/83/EG sowie der Verordnung (EG) Nr. 726/2004 (Text von Bedeutung für den EWR) OJ L 378, 27.12.2006, p. 1–19 (Aktuelle konsolidierte Version v. 28/01/2019)

[23] Richtlinie 2001/83/EG des Europäischen Parlaments und des Rates vom 6. November 2001 zur Schaffung eines Gemeinschaftskodexes für Humanarzneimittel. OJ L 311, 28.11.2001, p. 67–128

[24] Mahler V, Mentzer D, Bonertz A (2020) Allergen Immunotherapy (AIT) in Children: a vulnerable population with its own rights and legislation. Summary of EMA-initiated Multi-stakeholder Meeting on Allergen Immunotherapy (AIT) for Children, held at Paul-Ehrlich-Institut, Langen, Germany, 16.1.2019. Clin Transl Allergy 10:28. 10.1186/s13601-020-00327-w 10.1186/s13601-020-00327-w PMC7325268 32612805

[25] European Medicines Agency (EMA). (2015). EMA/PDCO Standard Paediatric Investigation Plan for Allergen Products for Specific Immunotherapy (EMA/PDCO/737605/2009) (Revision 4).

[26] Pfaar O, Agache I, Bergmann KC (2020). Placebo effects in allergen immunotherapy—an EAACI Task Force Position Paper. Allergy.

[27] Klimek L, Bachert C, Pfaar O (2019). ARIA-Leitlinie 2019: Behandlung der allergischen Rhinitis im deutschen Gesundheitssystem. Allergo J Int.

[28] AeDA/DGAKI (2018). Insektengift-Immuntherapie und Kontraindikationen. Fachinformation oder Leitlinie – was zählt?. Allergo J.

[29] Richtlinie 89/342/EWG des Rates vom 3. Mai 1989 zur Erweiterung des Anwendungsbereichs der Richtlinien 65/65/EWG und 75/319/EWG und zur Festlegung zusätzlicher Vorschriften für aus Impfstoffen, Toxinen oder Seren und Allergenen bestehende immunologische Arzneimittel. Amtsblatt EG L 142 vom 25.05.1989, S. 0014–0015

[30] Mahler V, Bonertz A, Weber G, Vieths S, Klimek L, Vogelberg C, Werfel T( (2019). Regulation von Allergenprodukten in Deutschland und behördliche Überwachung. Weißbuch Allergie in Deutschland.

[31] Gesetz über den Verkehr mit Arzneimitteln (Arzneimittelgesetz – AMG). Arzneimittelgesetz in der Fassung der Bekanntmachung vom 12. Dezember 2005 (BGBl. I S. 3394), zuletzt durch Artikel 16a Absatz 3 des Gesetzes vom 28. April 2020 (BGBl. I S. 960) geändert

[32] Bonertz A, Roberts GC, Hoefnagel M (2018). Challenges in the implementation of EAACI guidelines on allergen immunotherapy: A global perspective on the regulation of allergen products. Allergy.

[33] EDQM (2019). Eur. Ph. Monograph (No. 1063) on Allergen Products. European Pharmacopeia 10.0.

[34] EMA (2008). Guideline on allergen products: production and quality issues (EMEA/CHMP/BWP/304831/2007).

[35] EMA (2018). Concept paper on a guideline for allergen products development in moderate to low-sized study-populations (EMA/CHMP/251023/2018; Rheumatology/Immunology Working Party (RIWP).

[36] EMA (2020) Recommendations on common regulatory approaches for allergen products CMDh/399/2019, Rev.0 (July 2020). https://www.hma.eu/fileadmin/dateien/Human_Medicines/CMD_h_/procedural_guidance/01_General_Info/CMDh_399_2019_clean_Rev0_2020_07.pdf. Zugegriffen: 11. Sept. 2020

[37] Englert L, May S, Kaul S, Vieths S (2012). Die Therapieallergene-Verordnung. Hintergrund und Auswirkung. Bundesgesundheitsblatt.

[38] Mahler V, Bonertz A, Ruoff C (2019). What we learned from TAO—10 years of German therapy allergen ordinance. Allergo J Int.

[39] Paul-Ehrlich-Institut Allergene. https://www.pei.de/DE/arzneimittel/allergene/allergene-node.html. Zugegriffen: 14. Juli 2020

[40] Paul-Ehrlich-Institut Allergene. Verkehrsfähige Therapie-Allergene nach Therapieallergene-Verordnung (TAV) – Weitere Informationen. https://www.pei.de/DE/arzneimittel/allergene/therapie-verkehrsfaehig/verkehrsfaehig-node.html. Zugegriffen: 14. Juli 2020

[41] Pfaar O, Agache I, de Blay F (2019). Perspectives in allergen immunotherapy: 2019 and beyond. Allergy.

[42] Heiss S, Mahler V, Steiner R (1999). Component-resolved diagnosis (CRD) of type I allergy with recombinant grass and tree pollen allergens by skin testing. J Invest Dermatol.

[43] Matricardi PM, Kleine-Tebbe J, Hoffmann HJ (2016). EAACI molecular allergology user’s guide. Pediatr Allergy Immunol.

[44] Valenta R, Lidholm J, Niederberger V, Hayek B, Kraft D, Grönlund H (1999). The recombinant allergen-based concept of component-resolved diagnostics and immunotherapy (CRD and CRIT). Clin Exp Allergy.

[45] Muraro A, Roberts G, Halken S (2018). EAACI guidelines on allergen immunotherapy: Executive statement. Allergy.

[46] Vickery BP, Vereda A, Casale TB, PALISADE Group of Clinical Investigators (2018). AR101 Oral Immunotherapy for Peanut Allergy. N Engl J Med.

[47] Jones SM, Sicherer SH, Burks AW (2017). Epicutaneous immunotherapy for the treatment of peanut allergy in children and young adults. J Allergy Clin Immunol.

[48] Jensen-Jarolim E, Bachmann MF, Bonini S (2020). State-of-the-art in marketed adjuvants and formulations in allergen immunotherapy.

[49] Moingeon P (2012). Adjuvants for allergy vaccines. Hum Vaccin Immunother.

[50] de Souza Apostólico J, Lunardelli VA, Coirada FC, Boscardin SB, Rosa DS (2016). Adjuvants: classification, modus operandi, and licensing. J Immunol Res.

[51] EMA (2005). Guideline on adjuvants in vaccines for human use (EMEA/CHMP/VEG/134716/2004).

[52] EMA (2018). Draft guideline on clinical evaluation of new vaccines (EMEA/CHMP/VWP/164653/2005).

